# Size-Induced Constraint Effects on Crack Initiation and Propagation Parameters in Ductile Polymers

**DOI:** 10.3390/ma14081945

**Published:** 2021-04-13

**Authors:** Anja Gosch, Florian Josef Arbeiter, Silvia Agnelli, Michael Berer, Francesco Baldi

**Affiliations:** 1 Materials Science and Testing of Polymers, Montanuniversitaet Leoben, Otto-Gloeckel-Str. 2, 8700 Leoben, Austria; anja.gosch@unileoben.ac.at; 2 Dipartimento di Ingegneria Meccanica e Industriale, Università degli Studi di Brescia, Via Branze 38, 25123 Brescia, Italy; silvia.agnelli@unibs.it (S.A.); francesco.baldi@unibs.it (F.B.); 3 Polymer Competence Center Leoben GmbH, Roseggerstr. 12, 8700 Leoben, Austria; michael.berer@pccl.at

**Keywords:** material key curve, ABS, crack growth resistance, constraint, triaxiality, initiation parameter

## Abstract

Fracture mechanics are of high interest for the engineering design and structural integrity assessment of polymeric materials; however, regarding highly ductile polymers, many open questions still remain in terms of fully understanding deformation and fracture behaviors. For example, the influence of the constraint and specimen size on the fracture behavior of polymeric materials is still not clear. In this study, a polymeric material with an elastic plastic deformation behavior (ABS, acrylonitrile butadiene styrene) is investigated with regard to the influence of constraint and specimen size. Different single-edge notched bending (SENB) specimen sizes with constant geometrical ratios were tested. The material key curve was used to investigate differences in the constraint, where changes for small and large specimen sizes were found. Based on a size-independent crack resistance curve (J–R curve), two apparent initiation parameters (J_0.2_ and J_bl_) were determined, namely, the initiation parameter J_ini_ (based on the crack propagation kinetics curve) and the initiation parameter J_I,lim_ (based on an ESIS TC 4 draft protocol). It was found that J_0.2_ and J_bl_ could be used as crack initiation parameters whereby J_ini_ and J_I,lim_ are indicative of the onset of stable crack growth.

## 1. Introduction

Structural component design requires detailed information about the fracture behavior of a material in order to ensure the required safety. It is possible to predict the toughness or even the service life of a component with the use of fracture mechanical approaches by considering the influence of load, toughness, and inherent flaws in the material. In the case of a linear elastic material behavior (linear elastic fracture mechanics—LEFM), a single parameter is usually able to describe the fracture property (stress intensity factor, K, critical energy release rate, G, or crack tip opening displacement, CTOD) of a material; however, common applications of polymers often exceed the area of LEFM and show a material behavior where elastic plastic fracture mechanics (EPFM) must be considered. A typical result of EPFM is the so-called crack resistance curve (J–R curve, J-integral depending on the crack advancement Δa), which can be used to describe the fracture behavior of a material based on crack initiation and crack growth parameters. When external loads exceed a certain level, a crack starts to grow, which is typically expressed by the crack growth initiation parameter. This parameter characterizes only the onset of crack growth but provides no further information about the crack growth behavior of the material. The ability of the material to withstand crack growth is commonly known as crack growth resistance and is usually proportional to the shape and especially the slope of the J–R-curve [1].

In a fracture mechanical experiment on a plate with a defined crack, the stress state can vary along the crack front. A high constraint (triaxiality) is typically present in the middle of the plate (plane strain) and decreases close to the free surface (plane stress) [1]. Analogously, thickness varies within a component and this can lead to changing constraint levels, which can influence the fracture behavior [1,2,3]. As such, it is important to assess the crack initiation and crack growth parameters for changing specimen sizes.

Generally, the crack depth, specimen thickness, geometry of the crack and the loading situation can have a strong effect on the determined fracture parameters and refer to “constraint effects”, as shown in the literature [4]. The crack initiation parameter is generally found to not be highly sensitive to geometry changes for metals [4]. In contrast, the crack growth parameter usually displays a size-dependent behavior and is also influenced by the structural configurations [1,4]; however, the influence of specimen constraint on fracture parameters has been scarcely investigated in the field of polymers. Frontini et al. [5] studied the influences of different specimen configurations on the fracture parameters of polypropylene and found a dependency of fracture parameters on the chosen specimen thickness and width. Che et al. [6] proved the size-independent crack initiation behavior of polyvinyl chloride above a threshold thickness value of 10 mm, while some differences in crack growth were still visible. Previous research [7], dealing with the determination of the elastic plastic fracture behavior of up-scaled specimen sizes (increasing specimen size with identical geometrical ratios of width, thickness and initial crack lengths) of acrylonitrile butadiene styrene (ABS), showed strong size dependence of the determined fracture initiation parameters, while the first results indicated a size-independent crack growth behavior. Based on these results, it appears that it is not yet possible to determine a clear set of rules regarding constraint and corresponding fracture parameters of polymers under the conditions of EPFM. Subsequently, the current work aims towards a deeper understanding of aforementioned results obtained in [7] with regard to the influence of constraint and specimen sizes. 

To assess this up-scaling behavior of ABS, a testing procedure [8] from the ESIS TC4 (European Structural Integrity Society, Technical Committee 4 on polymers and polymer composites) was used for the determination of a pseudo-crack initiation parameter, J_I,lim_, and a parameter describing the crack growth process, m_s_. The applied testing procedure [8], named the TC4 LS method hereafter, is based on load separation theory [9] and requires only a few specimens for the evaluation of J_I,lim_ and m_s_. The TC4 LS method was originally proposed to strengthen the results of the commonly used multispecimen method [10], and its applicability to various types of polymers was already investigated in a round robin test under the direction of the ESIS TC 4 [11,12]. 

Especially for the crack initiation phase, represented by J_I,lim,_, a clear trend with increasing specimen size was found. The observed size-dependent behavior of J_I,lim_ was not expected, since a crack initiation parameter should be independent of the specimen size when all preconditions regarding specimen size are fulfilled (i.e., exceeding the minimum thickness for thickness independent fracture parameter) [1,4,6]. Interestingly, the presented J–R curve, determined via classical multispecimen approach [13], showed overlapping results and no indication of size dependence. Subsequently, this raised the question of whether the initiation values depend on the size of the specimen or if the applied procedure was inherently flawed; however, open questions about the level of constraint of the specimen size did not allow for a clear interpretation at that point.

Subsequently, the aim of the present study is to close this gap, by analyzing the effect of the constraint on the crack initiation and crack propagation phase for this material and specimen size in detail. As a starting point, the influence of specimen constraint is examined by resorting to the so-called calibration function. A common way to determine the calibration function is by the evaluation of the material key curve (normalized load, P_N_, as a function of the normalized plastic displacement, u_pl_/W) derived from the load separation principle (as shown in [2]). Further, to examine constraint effects in the crack growth phase, the stress states of specimens are deliberately changed by introducing side grooves. Finally, the gained knowledge about the specimen constraint is used for a clearer interpretation of the fracture process (i.e., crack initiation and crack growth) with a changing specimen size.

## 2. Theory and Calculation

The theoretical backgrounds for the methods used to evaluate constraint changes with increasing specimen size are described in detail in the next chapter. Furthermore, the applied procedure for the calculation of established crack initiation (J_bl_, J_0.2_ and J_ini_) and crack growth parameters (crack resistance curve, J–R curve) are given. These values are also used to validate the results of J_I,lim_, calculated via the TC4 LS method from prior work [7].

### 2.1. Constraint Effects in SENB Specimens

In the present study, two procedures are used to check the influence of constraint differences in the tested specimen sizes. In the first, the level of constraint is analyzed a posteriori via the material key curve construction for all the specimen sizes. As such, the resulting crack growth initiation values can be compared to their corresponding stress states. Additionally, the local stress states in specimens were deliberately altered a priori by introducing side grooves in selected specimens to examine differences in the crack growth phase.

#### 2.1.1. Determination of Constraint Level in the Crack Initiation Phase via the Material Key Curve 

The estimated material key curve is independent of the specimen geometry as long as the constraint is not modified. Therefore, the material key curve is a great tool to investigate changes in the constraint level during crack initiation. For the correct application of the material key curve, the load separation principle has to be verified beforehand, as presented for several polymers in the literature [11,14,15,16,17,18,19,20,21,22]. The material key curve is based on the load separation principle [9,23,24], in which the load, P, can be expressed as the product of two independent functions for a defined geometry, material and constraint (in the plastic region during a fracture test on a cracked specimen) [9]:

(1) $$P=G\;\left(\frac{a}{W}\right)\;H\left(\frac{u_{\mathrm{pl}}}{W}\right)$$

where G is the geometry function, H the material deformation function, a the notch length, W the specimen width, and u_pl_ the plastic displacement. The plastic displacement is given by [9]:

(2) $$u_{\mathrm{pl}}=u-\;C\left(\frac{a}{W}\right)\;P$$

where C(a/W) is the elastic compliance of the tested specimens. The load separation principle (as proposed in Equation (1)) is only valid for fracture tests on a cracked specimen with a defined geometry, material and constraint as discussed in [9,24]. For single-edge notched bending (SENB) specimens, beam-shaped specimens with a single edge notch under a three-point bending load, the geometry function, G, are defined by the following expression [24]:

(3) $$G=\;{\left(1-\frac{a}{W}\right)}^{\eta_{\mathrm{pl}}}$$

**Figure 1:**
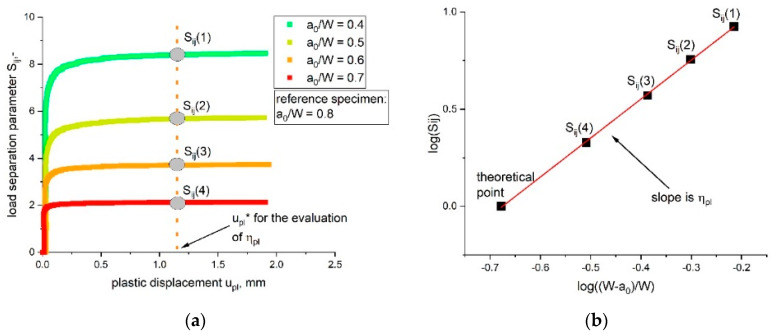
(**a**) Determined load separation curves (S_ij_ as function of u_pl_) of bN specimens with a changing a_0_/W ratio and the chosen limit value u_pl_*. (**b**) Evaluation of η_pl_ as the slope of the S_ij_ over the (W − a_0_)/W plot.

The geometry-independent plastic calibration factor, η_pl_, is given as two for SENB specimens in the literature [24]; however, the parameter η_pl_ is only valid if the load can be expressed in its separable form, like in Equation (1) [9,14]. This precondition can be verified experimentally by the separability parameter, S_ij_, as follows [9,24]:

(4) $$S_{\mathrm{ij}}=\;{\frac{P\left(a_{i}\right)}{P\left(a_{j}\right)}|}_{u_{\mathrm{pl}}}$$

where a is the remaining ligament length of the tested blunt notched specimens and P(a_i_) and P(a_j_) are the load values of blunt notched (bN) specimens with identical testing configurations and materials but various crack length over width ratios, a_0_/W (represented as a_i_ and a_j_ in Equation (4)). Based on these assumptions the presented separability parameter, S_ij_, can be simplified as follows [24]:

(5) $$S_{\mathrm{ij}}=\;{\frac{G\;\left(\frac{a_{i}}{W}\right)\;H\left(\frac{u_{\mathrm{pl}}}{W}\right)}{G\;\left(\frac{a_{j}}{W}\right)\;H\left(\frac{u_{\mathrm{pl}}}{W}\right)}|}_{u_{\mathrm{pl}}}=\;{\frac{G\;\left(\frac{a_{i}}{W}\right)\;}{G\;\left(\frac{a_{j}}{W}\right)\;}|}_{u_{\mathrm{pl}}}$$

where the material deformation function, H, is equal for the same material and ratio of the geometry function, G, representing S_ij_. As mentioned above, Equation (5) can be used to check the validity of the load separation parameter, which is expressed through the parameter η_pl_. The theory assumes that the geometry function G is constant for stationary crack experiments. Therefore, the separability curve (S_ij_-u_pl_ curve, with S_ij_ depending on u_pl_) of bN specimens with various a_0_/W ratios has to be determined for verification. One bN specimen needs to be defined as reference specimen, for example the specimen with the highest a_0_/W ratio (the highest a_0_/W value was 0.8 for our experiments). Afterwards, the parameter S_ij_ can be calculated with Equation (4) and the S_ij_-u_pl_ curve can be plotted (Figure 1a). The bN specimens display an almost constant value after the initial phase for all chosen a_0_/W ratios. In this plateau area of the S_ij_-u_pl_ curve, a fixed value of plastic deformation, called u_pl_*, is defined for the evaluation of η_pl_ (see Figure 1a). Subsequently, the values of the separability parameter S_ij_ at u_pl_^*^ can be plotted over the used notch length (in Figure 1b the ligament length over the width W − a_0_)/W of the tested bN specimens). For the used reference specimen (as aforementioned and in accordance with our experiments, in this example, a_0_/W is 0.8), a theoretical point is added, where S_ij_ is equal to zero. The parameter η_pl_ is evaluated as the slope of the curve shown in Figure 1b. The slope displays a constant value when a separable form of the load exists for a set of material, geometry and constraint. Hence, the validity of the load separation principle can be assumed for the investigated specimens. 

After the validation of the load separation principle, it is possible to determine the material key curve to examine constraint issues. The evaluation of the material key curve is based on the geometry function, H, evaluated from Equations (1) and (3) [24]:

(6) $$H\left(\frac{u_{\mathrm{pl}}}{W}\right)=\;\frac{P}{G\;\left(\frac{a}{W}\right)\;}=\;\frac{P}{{\left(1\;-\;\frac{a}{W}\right)}^{2}\;}$$

From Equation (6), the normalized load, P_N_, can be evaluated by normalizing the geometry function, H, as follows [24]:

(7) $$P_{N}=\;\frac{P}{B\;W\;{\left(1\;-\;\frac{a}{W}\right)}^{2}\;}$$

The material key curve is defined as the normalized load P_N_ plotted against u_pl_/W and it can provide information about changes in the constraint in front of the crack tip (see [20]). In this work, the material key curve is evaluated via stationary crack experiments on bN specimens, in which no crack growth was allowed. Previous studies showed good agreement between sN-based and bN-based material key curves [20,22]. Hence, a comparison of sN and bN specimens from the same material, tested with the same configurations and conditions can be used to determine differences in the specimen constraint. 

Generally, the material key curve is depending on the material deformation behavior and the geometrical constraint [20,23,24]. Previous research [20], dealing with the application of the material key curves on polymers, proposed a simplified relationship between P_N_, the constraint, L, the span length over width ratio, S/W, and the yield stress, σ_y_ [20]:

(8) $$L\;=\;\frac{P_{N\;}\;S}{\sigma_{s}\;W}$$

The application of Equation (8) is ideally limited to elastic plastic materials and assumes a fully yielded net section. In spite of this, based also on the results presented in [20], the material key curves were used to gain information regarding the constraint degree in the ABS specimens examined in the present paper.

#### 2.1.2. Changing the Constraint Level in the Crack Growth Phase by Testing Side-Grooved Specimens

To investigate constraint effects during actual crack growth, the testing of side-grooved specimens is a rather simple possibility. Side grooves change the zone of low constraint near the outer surface of a specimen and reduce the possibility of shear lip formation, which leads to a higher constraint level. The testing of side-grooved specimens, with higher constraint and stress within the specimen, can provide information about the sensitivity of the fracture process to overall constraint changes during crack propagation. 

### 2.2. Evaluation of Crack Initiation and Crack Growth Parameter 

After the aforementioned examination of the constraint in different specimen sizes, it was possible to further investigate and validate the determined fracture parameters. This knowledge may be used to interpret the results of the previous study [7], where the so-called pseudo initiation parameter J_I,lim_, based on the TC4 LS method [8], displayed dependence with an increasing specimen size. Furthermore, to not only validate these results with the examination of the level of constraint, but also to check for possible inherent flaws in the new testing procedure, established crack initiation and propagation values are also included in the study.

#### 2.2.1. Determination of the J–R Curve

**Figure 2:**
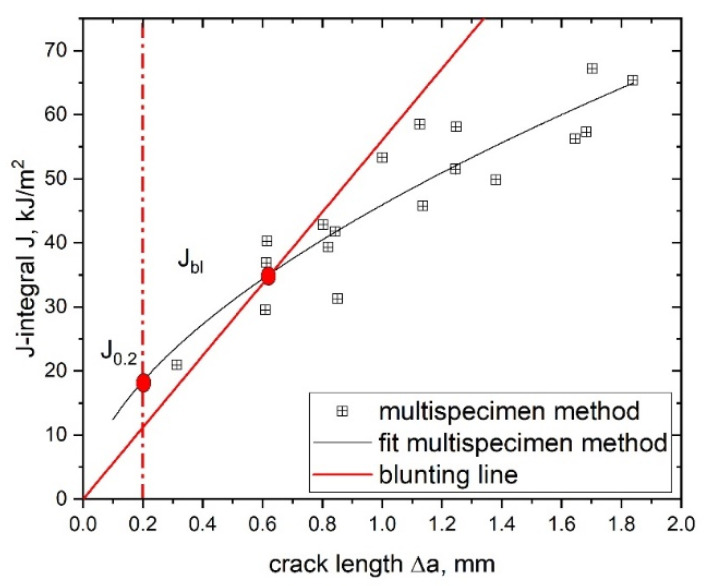
Evaluation of the initiation toughness parameters J_0.2_ and J_bl_ from the J–R curve (adapted with permission from [25]).

The most common method to determine fracture properties in an elastic plastic material is the so-called multispecimen method. For this approach, several identical specimens are tested with different crack advancement values. Subsequently, the energy necessary for the amount of crack growth, usually expressed via the J-integral, is plotted as a function of the produced crack advancement (Δa). In this work J–R curves were determined according to the ESIS TC-4 method [10] for each examined specimen size. According to the ESIS TC-4 multispecimen method, valid data points are limited by two critical Δa values (Δa_min_ and Δa_max_). The minimum Δa_min_ is fixed at 0.05 mm of crack advancement and the maximum Δa_max_ depends on the specimen size according to 0.1(W − a_0_). In the case of cracks with a length higher than 0.1(W − a_0_), the conducted measurements were corrected for high amounts of crack growth following the proposed formula in [9]:

(9) $$J={\;J}_{0}\left[1-\frac{0.75\;\eta -1}{W-a_{0}}\Delta a\right]$$

(10) $$J_{0}=\;\frac{{\eta U}_{c}}{B\;\left(W-{\;a}_{0}\right)}$$

where η is the geometry-dependent calibration factor and is 2 for SENB specimens, U_c_ is the corrected area under the load displacement curve (corrected for the amount of indentation according to [13]), B is the specimen thickness, W is the specimen width and a_0_ is the initial crack length. As a precondition for a successful application of the multispecimen procedure, the J–R curve data has to follow a simple power law routine [13]:

(11) $$J\;={\;c\;\Delta a}^{b}$$

where c and b are fitting parameters (see Figure 2). It is possible to determine both initiation and propagation values of the examined material by using this fitting curve.

#### 2.2.2. Determination of the Crack Initiation Parameters

Based on the calculated J–R curve, several fracture initiation parameters can be determined for a quantitative description of the investigated material; however, one of the most important parameters evaluated from the J–R curve is the technical crack initiation value, J_0.2_, at a crack advancement of 0.2 mm [13]. This crack advancement value was originally chosen since it was large enough to experimentally characterize real crack growth and small enough to be close to real initiation. The parameter J_0.2_ is widely accepted and used for the characterization of crack initiation in polymers [7,11,14,25,26,27]. The second initiation parameter used in this study is J_bl_, defined as the intersection of the blunting line with the J–R curve power law fit [13], where the blunting line is defined as follows:

(12) $$J=2{\;\sigma }_{y}\;\Delta a$$

where σ_y_ is the yield stress of the investigated material. A schematic J–R curve with both initiation parameters (J_0.2_ and J_bl_) is shown in Figure 2. 

**Figure 3:**
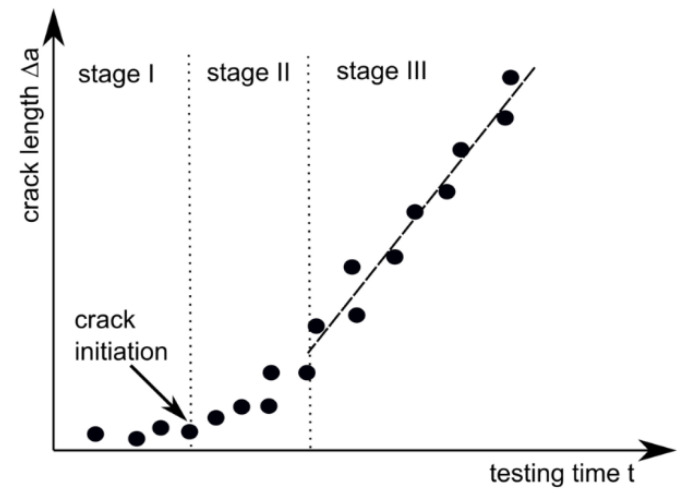
Crack propagation curve (crack length Δa marked as dots) for evaluating physical crack initiation with three characteristic stages: stage I (crack tip blunting), stage II (non-stationary stable crack growth) and stage III (stable crack growth).

A further crack initiation parameter used in this work is J_ini_, which is based on the crack propagation kinetics where the produced crack length, Δa, is plotted against the testing time of the experiment, t (Figure 3) [27,28,29]. Typical crack propagation kinetics curves exhibit three stages, where each stage represents a characteristic process in the crack growth mechanism during the process of a fracture. Stage I describes crack tip blunting and crack initiation, stage II describes non-stationary stable crack growth and stage III describes steady-state crack growth. The transition from stage I to stage II represents physical crack initiation, which makes this method especially interesting for the present study [27,28,29]. 

**Figure 4:**
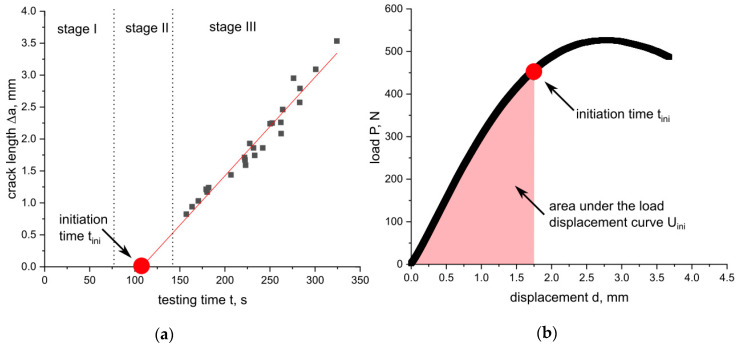
Crack propagation kinetics for the estimation of the initiation time t_ini_ (**a**) and evaluation of U_ini_ (area under the load displacement curve up to t_ini_) (**b**).

For the measured specimens in this study, no data points at a very low testing time were available (stage I), hence, the initiation time had to be verified with a slightly modified procedure (Figure 4a). The initiation time (t_ini_) was determined as the intersection between the linear data fit of stage III and the x-axis. Afterwards, the J-integral was calculated using the area under the load displacement curve, U, up to the displacement at t_ini_. The J-integral was calculated according to the recommended formula from the ESIS TC-4 procedure [10]:

(13) $$J_{\mathrm{ini}}=\;\frac{{\eta \;U}_{\mathrm{ini}}}{B\;\left(W-{\;a}_{0}\right)}$$

where η is the geometry-dependent calibration factor and is 2 for SENB specimens, U_ini_ is area under the load displacement curve up to t_ini_ (see Figure 4b) and corrected for the amount of indentation according to [13], B is the specimen thickness, W is the specimen width and a_0_ is the initial crack length.

## 3. Materials and Methods

The material and specimen geometries used in this work were identical to the previous study on the influence of size effects on fracture mechanical parameters [7]; however, the experimental setup for the evaluation of constraint effects featured several differences, which are described in detail in the following section.

### 3.1. Specimen Scale-Up 

**Figure 5:**
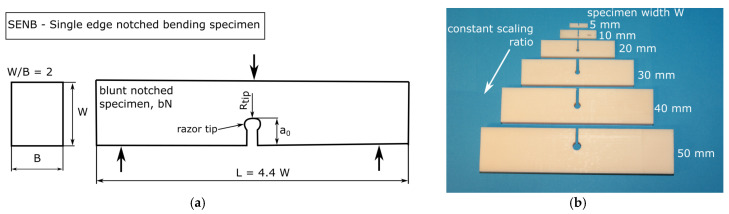
Blunt single-edge notched bending (SENB) specimen geometry and testing setup (**a**). Constant scale-up ratio for specimen width W with the manufactured blunt notched ABS specimens (**b**) (adapted from [7] with permission).

To analyse constraint effects with an increasing specimen scale, bN specimens of different sizes were manufactured. In addition to the bN specimens, sN specimens were made for the application of the multispecimen procedure in the same way as in the previous work [7]. The tested specimen geometry was of the SENB form, as shown for a bN specimen in Figure 5a, with a specimen up-scaling ratio of 10 (specimen width, W, from 5 mm to 50 mm as shown in Figure 5b). The chosen width to thickness ratio, W/B, as well as the length to width ratio, L/W, were kept constant for all specimen sizes according to [13]. The initial crack length over width ratio, a_0_/W, was constant for the sN specimens (0.6). Side grooves were added to three sN specimens where the sizes, W, were 10, 20, 30 and 40 mm in order to evaluate constraint differences. The manufactured side grooves had equal depth and showed a combined thickness reduction of 20% of the thickness B. For the bN specimens, the a_0_/W ratio varied from 0.3 to 0.8. 

### 3.2. Material 

The investigated material was ABS, which was supplied as extruded plates that were 1200 mm in length, 500 mm in width and 50 mm in thickness (identical to the previous study [7]). The high thickness of the plates was required to obtain a high up-scaling ratio (specimens with width values from 5 to 50 mm) for the manufactured specimens. The extruded plate is commercially available from Faigle Kunststoffe GmbH (Hard, Austria). The examined ABS showed a Young’s modulus (E) of 2200 ± 36 MPa, a tensile yield stress (σ_yt_) of 28.5 ± 0.1 MPa and a compressive yield stress (σ_yc_) of 47.4 ± 0.76 MPa (measured on specimens from the core of the plate) [7].

### 3.3. Specimen Preparation

**Figure 6:**
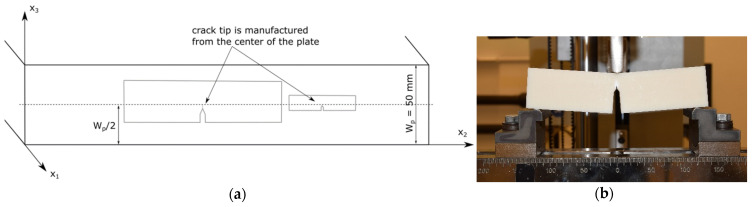
Scheme of the specimen manufacturing from the centre of the ABS plate to assure similar conditions near the crack tip (adapted from [7] with permission) (**a**); sN SENB specimen during testing (**b**).

The specimen preparation was made via cutting and milling. The blunt notch was introduced as a key hole notch with a size-dependent notch tip radius R_tip_ as listed in Table 1. As discussed in the previous work, material property variations between the edge and the centre of the thick plates were detected, which were caused by variations in the manufacturing conditions. To guarantee similar testing material behavior close to the round tip of all bN specimen geometries, a fixed thickness position of the plate was chosen as reference. This reference position is defined as half of the thickness of the plate (“W_p_/2”) as shown in Figure 6a.

### 3.4. Testing Procedures

Three-point bending tests on bN and sN specimens were conducted to characterize constraint issues in the fracture mechanical behavior of ABS. All mechanical tests were performed on a Zwick Universal Testing System (Zwick GmbH & Co. KG, Ulm, Germany), model Z010 or Z250 (see Figure 6b) with different load cells listed in Table 1. The measurements were taken in standardized conditions (23 °C air temperature, 50% relative humidity) with a constant loading rate of 1 mm/min. Detailed parameters concerning the experimental setup for the tested geometries are listed in Table 1. 

All mechanical fracture results (J-integral values) were corrected for the amount of indentation during the experiment. For this, the testing setup was changed to an indentation configuration and unnotched specimens were used to evaluate the indentation curve. Afterwards, this curve was subtracted from the measured load–displacement curves of the fracture mechanical specimens. Details concerning the indentation setup and procedure are given in [13].

## 4. Results and Discussion

### 4.1. Evaluation of Specimen Constraint

The evaluation of the constraint is helpful to understand the influence of the specimen size and the up-scaling behavior of fracture mechanical parameters. In the present study, the material key curve was used to identify changes in the specimen constraint influencing the crack initiation process. Therefore, the applicability of the load separation principle was verified beforehand since it is a precondition for the evaluation of the material key curve.

#### 4.1.1. Applicability of Load Separation Principle

**Figure 7:**
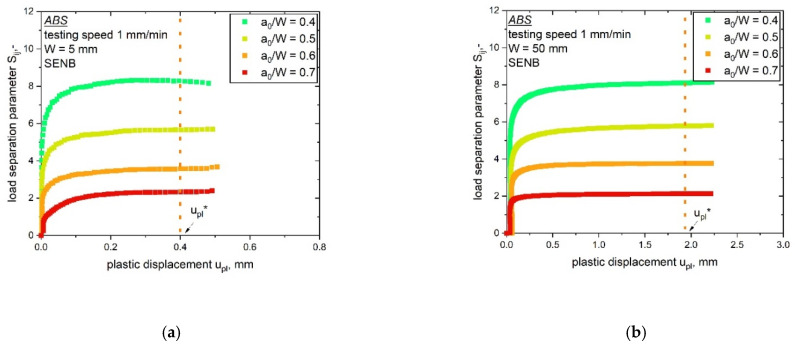
Decreasing load–displacement curves with increasing ratios of notch length over width a_0_/W for two tested bN ABS specimen sizes where W is 5 mm (**a**) and 50 mm (**b**).

Consequently, bN specimens with varying notch length (a_0_/W) were tested with varying displacement, which did not lead to crack growth initiation. The load–displacement curves of tested bN specimens are presented in Figure 7 for the smallest (W is 5 mm, Figure 7a) and the largest (W is 50 mm, Figure 7b) specimen sizes. 

**Figure 8:**
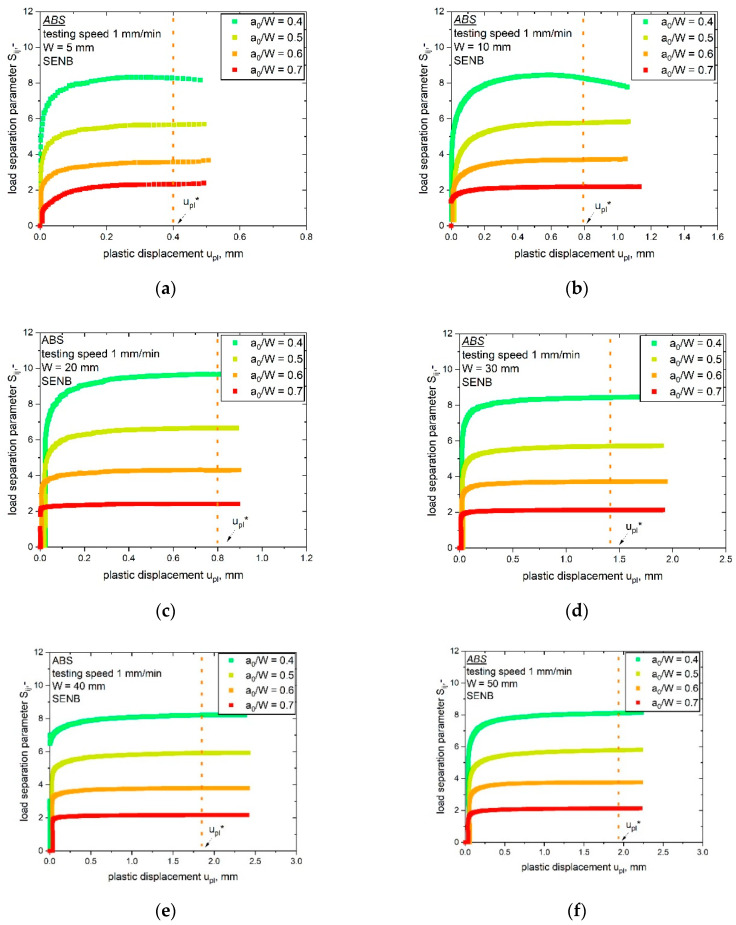
Evaluation of the parameter η_pl_ from S_ij_-u_pl_ curves determined from blunt notched specimens of ABS with various a_0_/W ratios and up-scaling specimen sizes (W values of 5 (**a**), 10 (**b**), 20 (**c**), 30 (**d**), 40 (**e**) and 50 mm (**f**)). The reference value of u_pl_ for the evaluation of η_pl_ (constant S_ij_ values) is marked as u_pl_* for every geometry.

For all geometries investigated, the measured forces increased with lower a_0_/W ratios. Furthermore, the measured load level was higher for a larger specimen size, as shown in Figure 7 for the specimens with W is 5 mm (a) and W is 50 mm (b). No crack growth was identified during the testing of the bN specimens, which allows the assumption of stationary cracks. This precondition is especially important for a correct verification of the load separation property, which was performed by the evaluation of the parameter η_pl_ as described in the theoretical part. The separability parameter, S_ij_, calculated from bN specimens with varying notch length over width ratio, a_0_/W, was calculated and plotted as a function of the plastic displacement, u_pl_, in Figure 8. As discussed in the experimental part, for the used reference specimen (a_0_/W is 0.8), a theoretical point was added where S_ij_ is equal to zero.

The S_ij_-u_pl_ curves were evaluated for all investigated specimen geometries (W values of 5 to 50 mm). Nearly all curves met the precondition of stationary cracks, as discussed in [14]. In the stationary crack experiments, the curves displayed a constant S_ij_ value (at high amounts of plastic displacement u_pl_) after the initial phase. For the first part of the S_ij_-u_pl_ curves in Figure 8, low u_pl_ values represent the initial phase of the experiment, in which the parameter η_pl_ was not defined. Hence, this phase was not of importance for validity; however, not all tested specimens displayed a stationary crack behavior. Specimens with the smallest notch length of 0.4 showed no clear plateau after the initial phase for the smallest two specimen sizes (W is 5 mm, Figure 8a and W is 10 mm, Figure 8b). This indicates that the crack growth in these two bN specimens was not completely prevented. Consequently, optical analyses were conducted to examine possible signs of crack initiation or crack growth close to the round notch tip of the bN specimen; however, no signs of crack growth were found in these two specimens. Therefore, they were included in the determination of η_pl_. The S_ij_ values used for the evaluation of the parameter η_pl_ are indicated in every plot in Figure 8 through the vertical line at u_pl_*. For all tested specimen geometries, the parameter η_pl_, determined as the slope of the plot shown in Figure 1b, was evaluated with its statistical coefficient R^2^ and is summarized in Table 2.

For all investigated specimen geometries, the value of the parameter η_pl_ was close to 2 with good statistical correlation described via the parameter R^2^ (Table 2). The estimated values were close to the theoretical value for this geometry (η_pl_ is 2 for SENB). Hence, the load separation validity was determined for all geometries examined in the present and previous works [7]. This first investigation of η_pl_ strengthens the previously determined results for the TC4 LS method regarding dealing with the specimen size effect [7]. Subsequently, the constraint issues for the up-scaled ABS specimens were evaluated. This was done by applying the material key curve method to the results of this section. 

#### 4.1.2. Determination of Specimen Constraint during Crack Initiation via Material Key Curve

**Figure 9:**
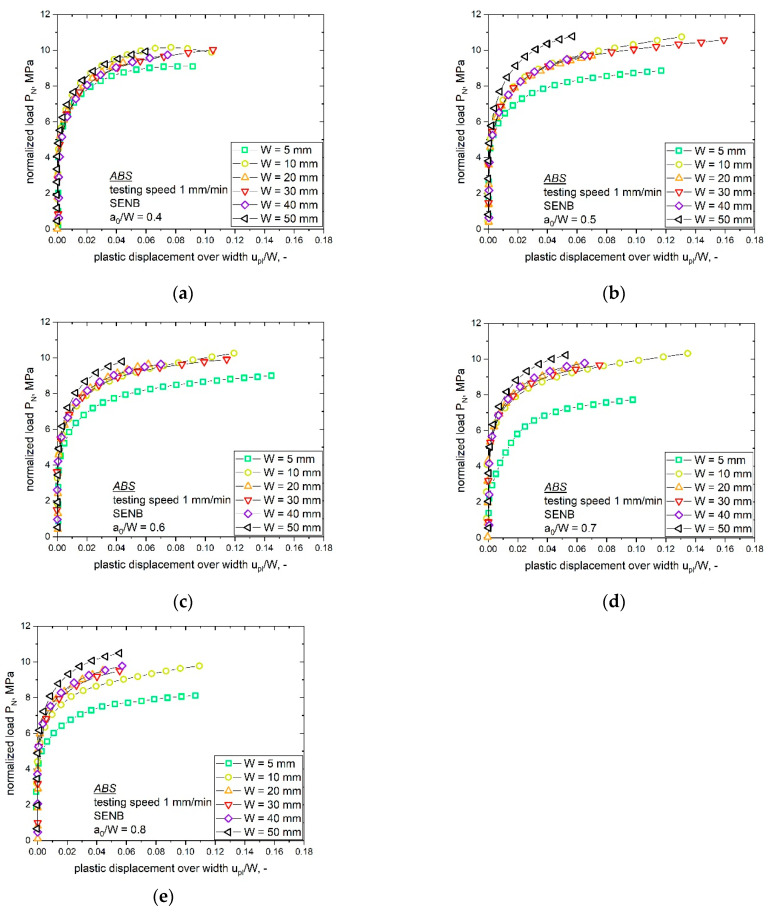
Material key curves for the investigated bN ABS specimens with different sizes (W of 5, 10, 20, 30, 40 and 50 mm) and varying a_0_/W ratios of 0.4 (**a**), 0.5 (**b**), 0.6 (**c**), 0.7 (**d**) and 0.8 (**e**).

The material key curves with varying bN specimen sizes (W of 5 to 50 mm) but similar notch lengths (a_0_/W ratio) were compared and are shown in Figure 9 (increasing a_0_/W ratio from Figure 9a–e). As discussed in the theory section, the normalized load P_N_ can be directly related to changes in the constraint as long as the testing conditions are constant (yield stress of the material, σ_y_, and span length over width ratio, S/W) [20]. Therefore, special attention was given to changes within the testing conditions. Small changes in σ_y_ related to the slightly different strain rates (see [7]) could be reasonably assumed to play a secondary role and were disregarded. Furthermore, the S/W ratio was kept constant. Hence, P_N_ can be used as an index for the constraint in front of the crack tip. 

The direct relationship between the material key curve and the level of constraint raised by the notch was only demonstrated for an ideally elastic plastic material, for which the material key curve was a horizontal line. In a real case, for a ductile polymer, the material key curve increases with increasing u_pl_/W value and if the displacement of the final point of the loading curve is sufficiently high then a plateau can be achieved. Even though it was not possible to determine a perfectly horizontal plateau region for the specimens examined within this work, a clearly different trend was presented by the material key curves of various sizes. In the present paper, the determined material key curves, at a given a_0_/W, flattened after the initial phase toward a plateau level. Therefore, the presented curves in Figure 9 can be seen as representative for the present constraint state. 

**Figure 10:**
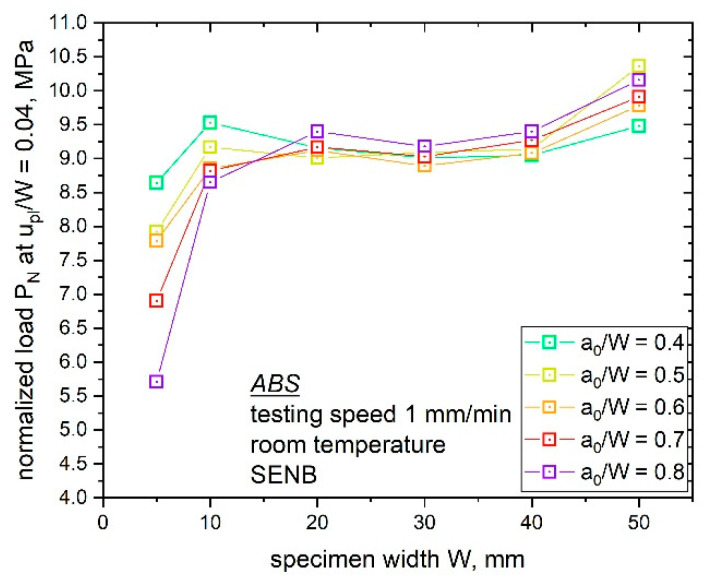
P_N_ at a fixed u_pl_/W ratio of 0.04 for every bN specimen size examined (W of 5, 10, 20, 30, 40 and 50 mm) with varying a_0_/W ratios (0.4 to 0.8).

The comparison of the different specimen sizes at a fixed a_0_/W ratio displays some deviations in the observed P_N_ values (Figure 9). Especially, with an increasing a_0_/W ratio, the material key curves of the smallest (W is 5 mm) and the largest specimen size (W is 50 mm) differed significantly. For specimens with the lowest a_0_/W ratio of 0.4 (Figure 9a), the observed material key curves showed low deviation between different specimen sizes. Hence, all ABS specimen sizes with an a_0_/W ratio of 0.4 displayed similar constraint situations in front of the notch tip. With an increasing notch length (a_0_/W ratio), trend changes and differences in the constraint values with increasing specimen sizes were observed, as shown in Figure 9b–d (a_0_/W ratio of 0.5, 0.6 and 0.7); however, for example, samples with W values of 10 to 40 mm and a a_0_/W ratio of 0.6 (Figure 9c), which is also the recommended a_0_/W ratio for the multispecimen procedure, showed similar material key curves, which indicates a similar crack tip constraint. The highest deviation in the values of P_N_ were observed for the largest specimen size (W of 50 mm). For the highest a_0_/W ratio of 0.8 (Figure 9e), higher deviation was observed but the trend was the same for the other configurations. These differences in the material key curves and subsequent stress states can be related to changes in the observed fracture initiation parameters. Hence, it is of high interest to evaluate these differences and consider geometry changes for accurate component design. To summarize this, the P_N_ values at a fixed ratio of u_pl_/W were evaluated (shown in Figure 10). For this, a u_pl_/W value of 0.04 was used since it was the highest level of u_pl_/W for which P_N_ data were available for all the specimen sizes and a_0_/W ratios. 

Two trends can be noticed in the material key curves and the compared P_N_ values in Figure 10, namely, the influence of the a_0_/W ratio for each investigated specimen size and the influence of the specimen size on P_N_ values at a fixed a_0_/W ratio.

Starting with the influence of varying notch length (a_0_/W ratio), the smallest specimen size (W is 5 mm) displayed the highest deviations in the calculated P_N_ values with an increasing a_0_/W ratio. As discussed in the previous section, the largest specimen size showed high differences in the obtained P_N_ values with a changing a_0_/W ratio. In contrast, the obtained P_N_ values for specimen sizes from W is 10 mm to 40 mm displayed no significant influence with a varying a_0_/W ratio (P_N_ values around 9 MPa were calculated for all notch lengths). By taking a closer look on the second influencing parameter shown in Figure 10, with a constant a_0_/W ratio and increasing specimen size, higher deviations in the obtained P_N_ values for higher a_0_/W ratios could be observed. For the lowest a_0_/W ratio of 0.4, small differences in the obtained P_N_ values (8.5 and 9.5 MPa) were observed. In comparison, the highest a_0_/W ratio of 0.8 resulted in P_N_ values between 5.5 and 10.25 MPa; however, specimens with the highest a_0_/W ratio could also be influenced by the small remaining ligament length, which can influence the full development of the plastic zone in front of the crack tip. 

The discussed constraint information is of great interest since size-dependent fracture behaviors were observed for up-scaled ABS specimens in a recently published work [7]. The previous study on sN ABS specimens was carried out at a fixed a_0_/W ratio of 0.6. It is obvious from Figure 9c and Figure 10 that almost no differences in the material key curve could be observed for specimen sizes between 10 to 40 mm for a fixed a_0_/W ratio; however, the material key curves for the small (W of 5 mm) and large (W of 50 mm) specimen size show significant differences. This supports the assumption of a changing constraint close to the crack tip for different specimen sizes. Since all specimen sizes from W is 10 to 40 mm displayed similar constraint states, it is of high interest to examine if the crack growth phase of these specimen sizes also exhibits a similar constraint state. 

### 4.2. Crack Growth Resistance Curve

#### 4.2.1. Crack Growth Resistance Curve from the Multispecimen Procedure

**Figure 11:**
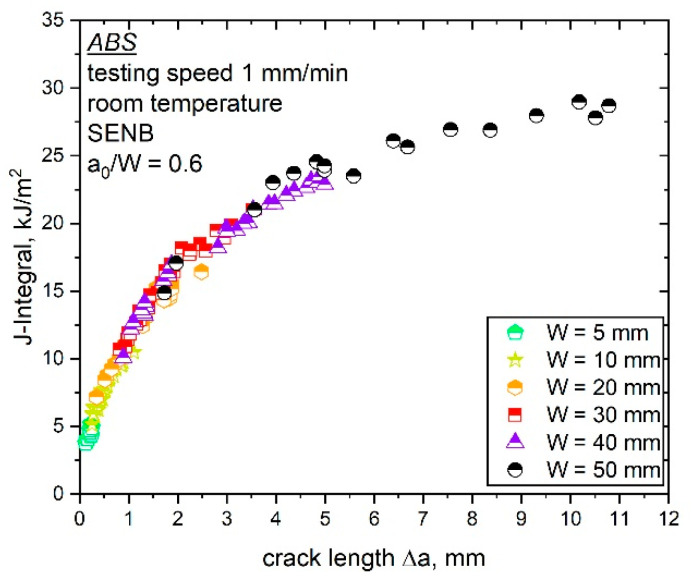
J–R curve for increasing specimen sizes (W is 5, 10, 20, 30, 40 and 50 mm) of ABS as determined following the ESIS TC 4 multispecimen procedure (adapted from [7] with permission).

In the previous study on size-dependent fracture parameters [7], it was not possible to precisely discuss the shape of the combined J–R curve due to a limited number of data points available at low values of Δa. This was due to the experimental setup of the load separation method, where high Δa values are required. Subsequently, more data points at lower Δa values were generated for the combined crack growth resistance curve in this work. Figure 11 shows the combined J–R curve of all specimen sizes (W is 5 to 50 mm) with these additional data points. The resulting crack growth resistance curve from Figure 11, including the additional test data, still displays one uniform curve. 

The J–R curve presented in Figure 11 displays one overlapping curve for all investigated specimen sizes of ABS; however, by taking a closer look on the curve shape of each evaluated J–R curve, it is no longer possible to describe the combined J–R curve (all data points of the investigated specimen sizes) via a power law fit (according to Equation (11)). Especially, for the smallest and largest specimens examined, a slightly deviating fracture behavior was observed, which was quantified via the application of a power law fit. The resulting fitting parameters c and b of each specimen size (listed in Table 3) show the expected deviations for the smallest (W is 5 mm) and largest specimen sizes (W is 50 mm), which is particularly noticeable in the case of the variation of parameter c. All examined specimen sizes in between (W of 10 to 40 mm) showed similar fitting parameters. Hence, a size-independent crack growth behavior could be assumed, where all examined specimen sizes (W of 10 to 40 mm) exhibited the same fracture resistance against crack growth. 

The specimen up-scaling method used in the present and previous studies [7], where all geometry parameters (B, L, a_0_) are dependent on the specimen width W, is rarely found in scientific work. This makes the comparison of the crack resistance curves determined here with results from literature challenging; however, evaluating plane stress and strain states with variations of the specimen thickness, B, at a constant specimen width, W, has been detailed in the literature [6,30]. The variation of specimen thickness is one of the most common procedures to investigate the influence of specimen size. With an increasing B, thickness-independent material constants can be determined (transition from plane stress to plane strain state). In contrast to this, the specimen width, W, usually has almost no influence on J–R curves as long as boundary conditions are not modified [30]. The presented simultaneous up-scaling procedure in this study, where B increases with an increasing W (fixed geometry ratio as in the present study), can also enable the calculation of the size-independent fracture parameters as shown in Figure 11. 

To increase the knowledge about the examined specimen sizes with similar fracture resistance curves (from W is 10 to 40 mm), the constraint situation was changed in the crack growth phase. Side-grooved specimens were tested for specimen sizes ranging from W is 10 to 40 mm and compared to the J–R curves presented in Figure 11. Side grooves change the zone of low constraint near the outer surface of a specimen and reduce the possibility of shear lip formation, which leads to a higher constraint level. The testing of side-grooved specimens with higher constraint and stress within the specimen can provide information about the sensitivity of the fracture process to the overall constraint changes during crack propagation. 

#### 4.2.2. Determination of Specimen Constraint during Crack Propagation via Testing of Side-Grooved Specimens

**Figure 12:**
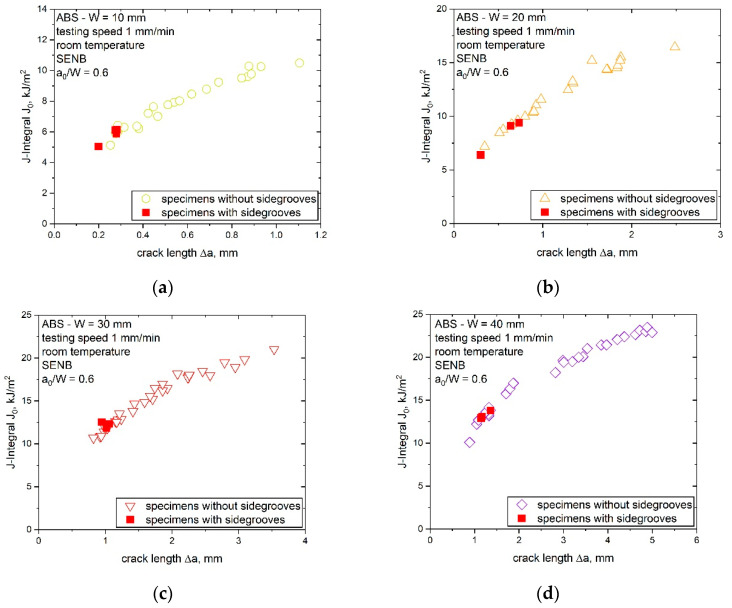
Resulting J–R curves of specimens tested with and without side grooves for increasing specimen sizes (W is 10 mm (**a**), 20 mm (**b**), 30 mm (**c**) and 40 mm (**d**), data points adapted from [7] with permission).

By the application of side grooves, constraint close to the edge changed and the constraint increased. Hence, it should be possible to confirm changes in the crack growth behavior by comparing the results of these specimens with the established J–R curves. Therefore, three side-grooved specimens of each specimen size (W of 10, 20, 30 and 40 mm) were tested according to the ESIS TC-4 multispecimen method [10]. The results from the side-grooved specimens were compared to the J–R curves of the previous study [7] and are shown in in Figure 12. 

The determined J–R curves in Figure 12 displayed no difference between specimens with and without side grooves, in contrary to the specimens with different sizes. The observed behavior denotes that the changing constraint in side-grooved specimens did not lead to a significant difference in the crack growth behavior. Similar behavior was also reported in a previous study on polypropylene specimens [5], where several specimen sizes with and without side grooves were compared. The results indicate that (for W of 10 to 40 mm) even if the constraint is artificially changed in the specimen, the crack growth behavior (at least as described by the J–R curve) does not change and a size-independent fracture behavior can be assumed. 

### 4.3. Fracture Initiation Parameters

#### 4.3.1. Initiation Toughness Parameter Determined from the J–R Curve

The initiation values of J_0.2_ for an increasing specimen size with the corresponding statistical coefficient R^2^ of the fitted J–R curve, determined according to the ESIS TC 4 multispecimen method, are listed in Table 3. For the evaluation of J_0.2_ values, the J–R curve of every specimen size examined was fitted (with the recommended power law according to Equation (11) [13]) and afterwards the J-integral was determined at 0.2-mm of crack growth. Hence, the evaluated J_0.2_ values were strongly dependent on the successful fitting of the J–R curve. Therefore, the statistical coefficient R^2^ of every fitted J–R curve is also listed in Table 3. Based on the significant differences in the shape of the J–R curve from the largest specimen size (W is 50 mm), its initiation parameters are highly questionable. The observed J_0.2_ values range from 4.5 kJ/m^2^ (for the smallest specimen, W is 5 mm) to 9.1 kJ/m^2^ (for the largest specimen, W is 50 mm). For the specimen sizes of 10 to 40 mm, the observed initiation parameters displayed nearly constant values with only a slight increase from 5.0 kJ/m^2^ to 6.3 kJ/m^2^; however, the smallest and largest specimen sizes displayed significant differences from the calculated initiation value. The resulting R^2^ of the fitting procedure displayed a low value for the smallest specimen size (W is 5 mm) due to the experimental difficulty of testing very small specimens (manufacturing accuracy, testing equipment, determination of the crack advancement using a light microscope). Hence, the determined crack initiation value, J_0.2_, for the smallest specimen size is also questionable. All other specimen sizes displayed R^2^ values from 0.936 to 0.984, which indicates that all data points could be fitted well with the applied power law. 

Furthermore, the initiation parameter J_bl_ was evaluated (listed in Table 3) as the intersection of the blunting line with the fit of each J–R curve. Therefore, the yield stress σ_y_ was used (28.5 MPa), which was investigated in the previous study [7]. J_bl_ displayed fracture initiation values from 2.4 kJ/m^2^ (W is 5 mm) to 4.2 kJ/m2 (W is 40 mm) which were significantly lower than the evaluated values for J_0.2_; however, both initiation values displayed a similar trend. Based on the multispecimen procedure [13], the lowest initiation value has to be taken as the initiation toughness parameter, which in this case is the blunting value J_bl_; however, J_bl_ depends on the successful fitting of the J–R curve, as discussed for J_0.2_. Both initiation parameters (J_0.2_ and J_bl_) refer to a region of the J–R curve that is quite far from the experimental data points used for its construction, especially for larger specimen sizes. Hence, they have to be interpreted as apparent values. Furthermore, the fitting regions (Δa range) differed for the examined specimen sizes. In consideration of this, it can be reasonably assumed that the values of the initiation parameter (J_0.2_ and J_bl_) could be influenced by computational effects, especially for the highest size examined (W of 50 mm). Further methods for the characterization of initiation parameters were conducted in order to improve the understanding of the present crack initiation behavior.

#### 4.3.2. Initiation Toughness Parameter J_ini_

**Figure 13:**
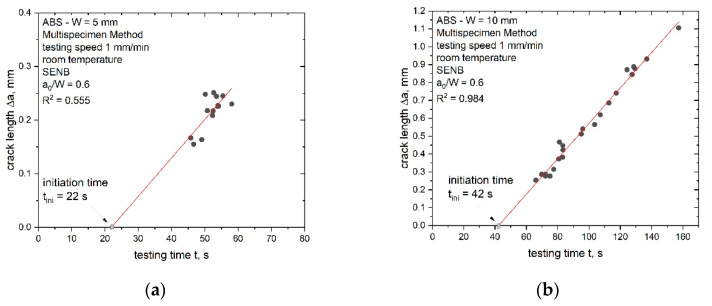

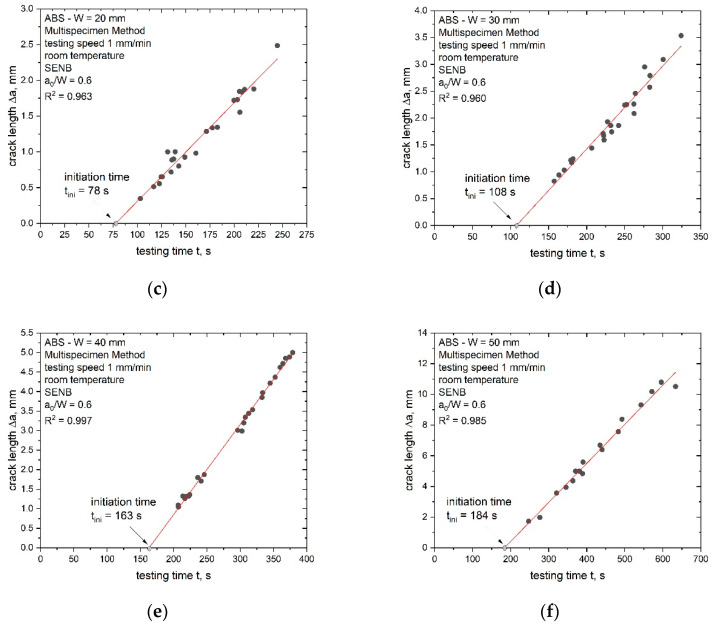
Determination of the crack initiation time, t_ini_, via the crack propagation kinetics curve (produced crack length Δa (black dots) depending on the testing time, t) for all investigated specimen sizes of ABS (W of 5 (**a**), 10 (**b**), 20 (**c**), 30 (**d**), 40 (**e**) and 50 mm (**f**)).

The calculation of the crack propagation kinetics curve, where Δa is plotted against the testing time, t, is an additional method for the investigation of entire fracture processes. It is possible to evaluate the parameter J_ini_ based on the crack propagation kinetics curve which represents crack initiation. Therefore, the crack initiation time, t_ini_, is required beforehand. The determined crack propagation kinetics curve with its fitting curve and the estimated initiation times, t_ini_, are shown in Figure 13 for the ABS specimens with different sizes. 

The limited data points at low testing times, representing the blunting process (stage I) and crack initiation (stage II), led to a slightly modified experimental procedure for the determination of t_ini_. The initiation time was estimated as the intersection of the linear fit of the available data points from stage III (representing crack growth) with the x-axis (details in the experimental section). The initiation time, t_ini_, increased with increasing specimen sizes and for all specimen sizes, with the exception of W = 5 mm, and displayed good R^2^ values for the applied linear fit as shown in Figure 13. In the case of the smallest specimen size (W of 5 mm), R^2^ showed a very low value of 0.55 (Figure 13a). Subsequently, the value of t_ini_ for W = 5 mm should be considered carefully. For the other investigated geometries (W of 10 mm to 50 mm), a good application of a linear fit was possible. Additionally, the crack growth speed in stage III was determined and the increase was found to be small over the whole scaling range (increase from 0.4 to 1.5 mm/min from the smallest to the largest specimen size). 

**Figure 14:**
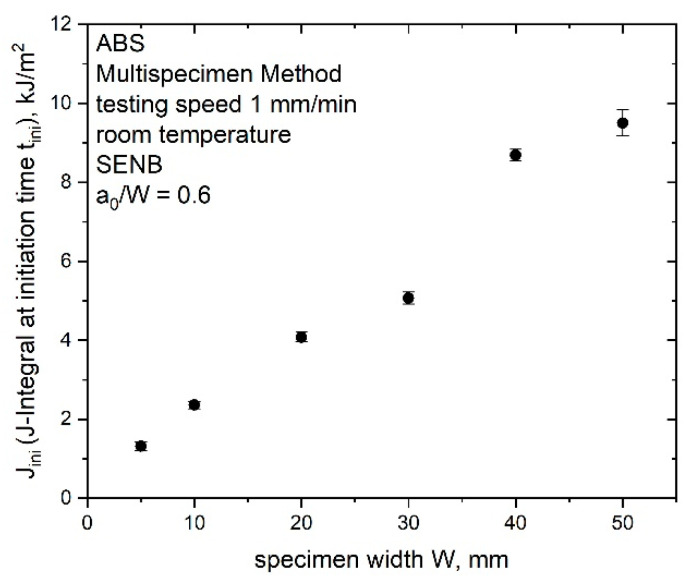
Growing initiation toughness value J_ini_ (J-integral at the initiation time t_ini_) for increasing specimen sizes (W is 5, 10, 20, 30, 40 and 50 mm) of ABS.

For a better comparison to the other crack initiation parameters, J_ini_ (J-integral at the initiation time t_ini_) was calculated and is presented in Figure 14 for the up-scaled specimens of ABS. The physical crack initiation (J-integral at initiation time t_ini_) increased with an increasing specimen size (from 0.8 kJ/m^2^ for W is 5 mm to 9.5 kJ/m^2^ for W is 50 mm) and displayed low standard deviation for all measured specimen sizes. In comparison with the evaluated J_0.2_ values (Table 3), the calculated J_ini_ values were smaller and exhibited a strongly size-dependent behavior. Size-dependent initiation toughness values were also found in literature [5], where methods from LEFM (stress intensity factor “K_Q_^”^, “K_max_”) were used to describe the elastic part of the J–R curve. Similar to results in this study, the linear elastic fracture parameters (“K_Q_^”^, “K_max_”) increased with increasing specimen size. 

#### 4.3.3. Comparison of Crack Initiation Parameters

**Figure 15:**
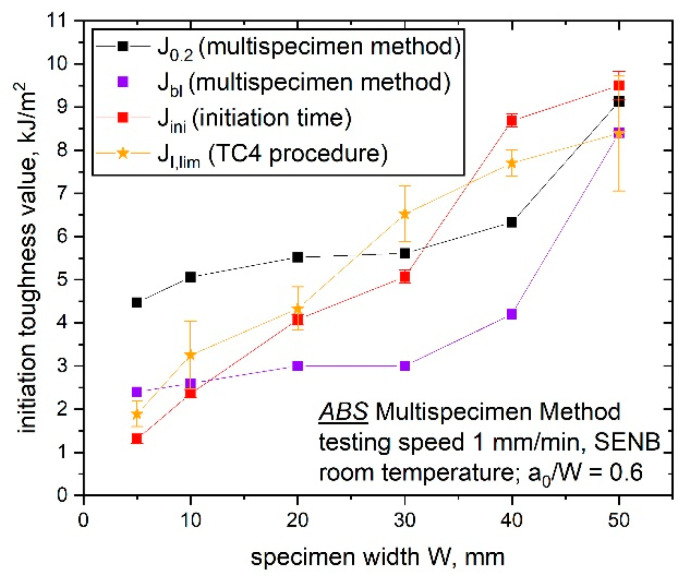
Comparison of initiation toughness parameters J_0.2_ (multispecimen procedure), J_bl_ (multispecimen procedure) J_ini_ (based on the initiation time t_ini_) and J_I,limi_ (ESIS TC 4 draft protocol) for increasing specimen sizes of 5, 10, 20, 30, 40 and 50 mm (adapted from [7]).

In the present study, additional tests on up-scaled sN ABS specimens were performed to increase the completeness of the J–R curve data from the previous study [7]. In this previous work, the characterized J–R curve displayed size-independency, whereby the initiation parameter (J_I,lim_ from the ESIS TC 4 draft protocol [8]) exhibited a strongly size-dependent behavior. A size-independent crack resistance curve for specimen sizes of 10 to 40 mm was confirmed by the additional data points measured following the ESIS TC 4 procedure; however, the calculated apparent initiation toughness parameters J_0.2_ and J_bl_ (based on the ESIS TC 4 multispecimen procedure) and J_ini_ (based on the crack propagation curve and t_ini_) displayed different fracture initiation behavior for the examined specimen sizes (size-dependent crack initiation parameter). For the sake of comparison, all calculated initiation toughness parameters (J_0.2_, J_bl_, J_ini_ and J_I,lim_) are shown in Figure 15. 

The initiation value J_0.2_ displayed the highest values for small specimen sizes as compared to the other assessed initiation parameters. For the specimen sizes of 10 to 40 mm, J_0.2_ displayed only a slight increase and indicated a plateau where the smallest (W is 5 mm) and the largest (W is 50 mm) specimen sizes displayed some differences. The observed fracture initiation behavior of J_0.2_ is explained by the observed constraint differences for the smallest and largest specimen sizes and a simply computational effect arising with the chosen fitting range. J_bl_ showed a similar trend as J_0.2_ (Figure 15); however, it showed the lowest fracture initiation values and therefore a representative initiation toughness value according to the multispecimen procedure [13]. For both parameters (J_0.2_ and J_bl_), no standard deviation was added in this plot since these values were determined by the intersection of the J–R fitting curve. It has to be noted that J_bl_ is not only influenced by the computational effect of the fitting range, but there is also the additional dependency on the evaluated σ_y_. Since small changes in σ_y_ are assumed to play a secondary role [7], the influence on the initiation value can also be negligible; however, for a successful evaluation of an initiation value this aspect has to be kept in mind. 

The calculated J_ini_ values (based on the initiation time t_ini_) depicted a continuously increasing initiation value with an increasing specimen size and the values were in the same range as the observed J_I,lim_ data from the previous research [7]. The standard deviations for the J_ini_ values were small in comparison to the determined values of J_I,lim_ [7]. These deviations between the initiation values, which were based on the multispecimen method (J_0.2_ and J_bl_) and the other two initiation parameters (J_ini_ and J_I,lim_), give rise to the assumption that these parameters mark two different stages during the fracture process. The estimated apparent values from the J–R curve (J_0.2_ and J_bl_) represent crack initiation, whereby J_ini_ and J_I,lim_ mark the beginning of stable crack growth. It is difficult to define crack initiation for ductile polymers since it is a continuous process from blunting to crack advancement [19]; however, in this work, crack initiation was defined as the early starting point of a progressive process of crack propagation. Typically, cracks started to grow at the notch tip from the inner region of a specimen. Furthermore, the stable crack growth phase is related to a fully developed crack front along the whole thickness and a constant crack propagation rate. This was not experimentally checked within this work and is a topic for future work. The assumption that J_0.2_ and J_bl_ represent crack initiation and J_ini_ and J_I,lim_ mark the beginning of stable crack growth was based on the differences in the used experimental approach of the presented parameters. As discussed in the previous section, J_ini_ was calculated by the intersection between the linear data fit of stage III (area of stable crack growth) and the x-axis. Hence, the influence of the blunting phase is not represented in the estimation of J_ini_, which strengthens the hypothesis that J_ini_ marks the point of stable crack growth instead of crack initiation. The parameter J_I,lim_ was suggested as a parameter for indicating stable crack growth, since a fixed value in the normalized load separation curve is defined as J_I,lim_ in the ESIS TC 4 procedure [8]. This fixed value is defined after the blunting phase and at the beginning of stable crack growth. A slight overestimation of the crack initiation by the parameter J_I,lim_ and the optical analysis of the fracture initiation has already been discussed in the literature [19]. 

Generally, fracture initiation and the point of stable crack growth must not be at the same level during a fracture experiment, which is also shown in Figure 15. For small specimen sizes, crack initiation (marked by J_bl_) and the point of stable crack growth (marked by J_ini_ or J_I,lim_) nearly occurred at the same J-value; however, for increasing specimen sizes the crack initiation values were significantly lower compared to the point of stable crack growth. This can be explained by the increasing specimen thickness and the particular nature of crack initiation, which is typically a progressive process characterized by a slow development over the whole crack front [19]. Based on these new findings, the parameter J_0.2_ was above the limit value of stable crack growth for small specimen sizes and below for large specimen sizes. Therefore, it is suggested to adapt the fixed initiation value J_0.2_ to a more geometry-dependent value to take changing specimen sizes into account. 

## 5. Conclusions

It is necessary to acquire detailed knowledge of the up-scaling relationships of fracture parameters (crack initiation and crack growth) and the constraint (crack tip triaxiality) to design complex components. In the case of polymers, the influence of scaling the specimen size on the elastic plastic fracture parameter has scarcely been investigated. Basic relationships like the influence of increasing specimen size or changing ligament length have already been discussed [5,6]; however, the present work gives insight into the dependency of the constraint and fracture behavior on the specimen size (SENB specimens with a maximum up-scaling ratio of 10) of ABS.

Changing constraint levels in the crack initiation and crack growth phase were evaluated by the application of the material key curve method for the smallest (W is 5 mm) and the largest (W is 50 mm) specimen sizes; however, all investigated specimen sizes in between (W from 10 to 40 mm) displayed a similar constraint for the crack initiation and crack propagation. With regard to the crack propagation behavior, it was found that the introduction of side grooves and a subsequent change in the overall stress state showed no influence on the results when compared to the J–R curves of non-grooved specimens. 

The resulting J–R curves showed one overlapping curve for all tested specimen sizes of ABS; however, for the smallest and the largest specimen sizes, some changes in the fitting parameters were detected, which supports the results of changing constraint levels for these two specimen sizes. The changing constraint state for small and large specimens is also represented in the apparent initiation parameters based on the J–R curve (J_0.2_ and J_bl_).

Furthermore, the influence of the specimen size on the fracture initiation was investigated. Therefore, four parameters were analyzed and compared in detail:

- J_0.2_ (apparent), which is based on the technological evaluation of the J–R curve [13] and displayed slowly increasing initiation values for specimens with W values of 10 to 40 mm.
- J_bl_ (apparent), which is also based on the technological evaluation of the J–R curve [13] and displayed the lowest initiation values and a similar behavior to J_0.2_.
- J_ini_, which is based on the initiation time, t_ini_, and displayed increasing crack initiation values with increasing specimen size where small deviations were detected for changing constraint states.
- J_I,lim_, which is based on the ESIS TC4 LS method and displayed similar results as J_ini_, thus supporting the size-dependent fracture initiation behavior (initiation parameters increase with increasing specimen size).

The contrary behavior of the four initiation parameters can be explained by a closer look into the evaluation and the experimental approach, where J_0.2_ and J_bl_ are more technological parameters describing the crack initiation. J_ini_ and J_I,lim_ were based here on the physical crack initiation and mark the point of stable crack growth. In spite of the apparent characteristics attributed to J_0.2_ and J_bl_, the results of the investigation suggest the use of the initiation parameters J_0.2_ and J_bl_ for material ranking and comparison due to the low differences in the resulting values with different geometries. On the contrary, the parameters J_ini_ and J_I,lim_ are of high interest regarding evaluating points of stable crack growth.

In the future, further investigations of changing constraint state for small and large specimens are planned. Furthermore, the experimental results found here will be compared with numerical simulations to gain more information about the stress state and constraint close to the crack tip. In addition, the crack growth process has to be examined in detail and compared with the two types of initiation parameters found in this study.

## Figures and Tables

**Table 1:** Detailed information on the experimental setup and the crack tip radius of the bN specimen for all specimen sizes examined.

Specimen Width W (mm)	Span Length S (mm)	Roller Radius (mm)	Load Cell Capacity (kN)	Crack Tip Radius of bN Specimen (R_tip_, mm)
5	20	1	1	0.5
10	40	3	10	1
20	80	3	10	2
30	120	5	10	3
40	160	5	10	4
50	200	10	10	5

**Table 2:** Values of η_pl_ with the corresponding statistical coefficient R^2^ for all tested specimen geometries (W is 5, 10, 20, 30, 40 and 50 mm).

Specimen Width W	Parameter η_pl_	R^2^
[mm]	-	-
5	1.98	0.999
10	2.03	0.999
20	2.03	0.999
30	2.01	0.999
40	1.94	0.999
50	1.94	0.999

**Table 3:** Power law fitting parameter (c and b according to Equation (11)) of the J–R curve with the corresponding statistical coefficient, R^2^, and initiation toughness value, J_0.2_, for increasing specimen sizes (W of 5, 10, 20, 30, 40 and 50 mm) of ABS specimens as determined following the ESIS TC 4 multispecimen method.

Specimen Width (W, mm)	Parameter c (Power Law Fit, kJ/(m^2^mm^b^))	Parameter b (Power Law Fit, kJ/(m^2^mm^b^))	R^2^	Crack Initiation (J_0.2_, Apparent, kJ/m^2^)	Crack Initiation (J_bl_, Apparent, kJ/m²)
5	8.7	0.41	0.718	4.5	2.4
10	10.4	0.45	0.972	5.0	2.7
20	11.3	0.45	0.978	5.5	3.0
30	11.9	0.47	0.969	5.6	3.0
40	12.3	0.41	0.984	6.3	4.2
50	14.6	0.29	0.936	9.1	8.4

## Data Availability

The data presented in this study are available on request from the corresponding author. The data are not publicly available due to ongoing research.
